# Co-ensiled rice straw with whole sugar beet and its effect on the performance of lactating cows

**DOI:** 10.1007/s11250-024-03945-9

**Published:** 2024-05-23

**Authors:** Fawzy Mohamed Abo-Donia, Hanim Abdelrahman Elsheikh, Ayman Mohamed Hosny Esh, Mohamed Ahmed Hassan El-Shora, Yasser Mabrouk Mandour Eldiahy

**Affiliations:** 1 By-product Utilization Research Department, Agriculture Research Center (ARC), Animal Production Research Institute (APRI), Nadi El-Said St, Dokki, Giza, 12611 Egypt; 2 Biotechnology Dept, Sugar Crops Research Institute, Agricultural Research Center, Giza, Egypt; 3 Cattle Breeding Res. Dept, Animal Production Research Institute, Agricultural Research Center, Dokki, Egypt

**Keywords:** Ensiled rice straw, Whole sugar beet, Milk production, Digestibility

## Abstract

This study investigated the effect of co-ensiled rice straw (RS) with whole sugar beet (SB) on lactating cows’ performance. Ensiled rice straw (ERS) as control (CGS) was incorporated with immersed corn grains (CG) for 24 h, while the 2nd and 3rd ensiled RS (LSB and HSB) contained SB substituted of 50 and 100% of CG on an energy basis (total digestible nutrients, TDN), respectively. In the experimental diets, D1, D2, and D3, which include CGS, LSB, and HSB provided *ad-libitum*, respectively, while a concentrated feed mixture (2% of body weight) was offered. The population of lactic acid bacteria was slightly higher with fed HSB, relative to LSB and CGS. The OM, CP, EE, NFC, and TCH contents of CGS were slightly higher than LSB and HSB, while the opposite happened with the aNDFom, and ADFom contents. The digestibility of DM, OM, aNDFom, and ADFom of the D3 group was higher (*P* < 0.05) than in D1 and D2. The D3 recorded the highest values (*P* < 0.05) of silage consumption, and palatability. Milk production, fat-corrected milk (FCM), and energy-corrected milk (ECM) were (*P* < 0.05) higher for cows fed D3 compared with D1 and D2. Fat, protein, lactose, and total solids were trending on the same track. The feed conversion ratio (FCR) of cows fed diet D3 was better than cows fed D1 diet. The level of glucose in the blood increased (*P* < 0.05) significantly with feeding on HSB than LSB, which was significantly (*P* < 0.05) higher compared to CGS. In conclusion, co-ensiling of RS with the whole SB plant consider a good method to improve its nutritional value.

## Highlights

Co-ensiled sugar beet with urea-treated rice straw reduced the contents of OM, CP, EE, NFC, and TCH while the opposite appeared in aNDFom and ADFom.

The incorporation of high-level sugar beet with ensiled rice straw results in a slight increase in the LAB number

The incorporation of sugar beet with ensiled urea-treated rice straw improved the nutritional value and feed intake

Co-ensiling of RS with the whole SB plant improved palatability, biodegradability, and nutrient digestibility

## Introduction

Despite the huge production of RS (ranging from 2.7 to 8 tons/hectare), the quantities used as animal feed are very little (Oladosu et al. 2016). A large amount of RS is burned directly in the field causing obvious air pollution. Fiber digestibility is consequential in a diet that includes low-quality roughages that are high in fiber, whether it is a grazing system, or if livestock is receiving low-grade quality hay (Nguyen et al. 2020). By increasing fiber digestibility, ruminants can efficiently obtain more additional nutrients from low-quality roughages (Salama et al. 2007). The limited use of rice straw in feeding ruminants is mainly due to composed of cellulose, lignin, unbalanced and low nutritional contents, low voluntary intake, and low digestion. Many attempts have been made to increase RS digestibility by the use of various treatments such as urea treatment, and fungus treatment (Nguyen et al. 2020).

Prior studies have shown that rice straw can ensiling, but may not produce high-quality silage due to low water-soluble carbohydrate (WSC, 29.7 g/kg DM) content and high levels of dry matter content (Li et al. 2010). Feeding sugar-based energy sources to livestock may have some metabolic benefits compared to feeding starch-based energy sources, especially at moderate levels (Evans and Messerschmidt 2017). However, little research has been conducted with ensiling sugar beets, much of the research has utilized sugar beet pulp. Sugar beets contain a considerable amount of unrestricted energy, and available energy has been displayed to decrease the pH of a silage combination (Poorkasegaran and Yansari 2014; Oladosu et al. 2016; Beauchemin 2006). A rapid drop in pH (3.8-4.0) is the most effective mode of inhibiting the enzymes that degrade protein, and fiber throughout the ensiling process (Li et al. 2010; Ni et al. 2018). Moreover, successfully ensiled SB with roughages reduces the negative effects of oxalate in beet leaves (Beauchemin 2006). Whereas, Hokama et al. (2000) concluded that lactic acid bacteria in silage use oxalate as a carbon source in nutrient-poor media. Consequently, The purpose of this study was to investigate the feeding of co-ensiled rice straw with whole sugar beet on the performance of lactating cows.

## Materials and methods

The study was conducted according to a cooperation protocol between the Animal Production Research Institute and Sugar Crops Research Institute of the Agricultural Research Center, at Sakha Experimental Station (Kafr El-Shaikh Governate, Egypt).

### Ethics statement

The study procedure and ethics were approved by Care the Research Committees in Animal Production Research Institute (Code No. APRI/ARC/2-2-2-4-2-9).

### Bundling of experimental ingredients and ensiling rice straws

The used RS was obtained from the supplier merchants of straw, who collects straw from farmers and then chopped it to 2–3 cm in length. The whole SB (beet-root and tops) was obtained at yield harvest from Sugar Crops Research Institute, at Sakha Agricultural Experiment Station. The beetroots were washed manually without wetting the leaves to get rid of traces of soil residue on the roots, then it was spread for a day on burlap sacks above a layer of rice straw 25 cm to get rid of excess water and prevent contamination. Before the ensiling process, beets are crushed via a small machine for 3–5 cm to incorporate with RS, and the DM content stood (at 24.31%). Rice straw was treated with urea solution (10 kg of urea dissolved in 300 L of water per ton of RS on a DM basis) and then divided into three piles. In 1st pile, ensiled RS as control (CGS) was prepared by adding 81.96 kg CG (90.96% DM) after being immersed in water for 24 h and then minced using a local mill to incorporate per each ton ureated-rice-straw (URS). In the 2nd pile, incorporated low-level beets (150 kg, LSB) plus 40.98 kg CG per ton URS. The 3rd pile included a high level of SB (HSB) consisting of, 300 kg SB per ton URS.

A plastic sheet was spread on a flat and dry ground surface and all material was mixed well by hand and then laid pressed in a stack by a tractor. The top was covered with plastic and sealed all around with 10 cm of fine soil, then above were placed old tires or any other suitable objects to prevent the top cover from being blown away by the wind. Samples were directly gathered before and after 45 d of ensiling from six different places of each pile individually to represent the pile, and then mixed well to pick kg as a representative sample. All samples of experimental silage were stored at -20 °C for the analysis of fermentation quality and chemical analysis.

### Animals, feeding, and digestibility trials

Nine mixed multiparous Friesian crossbred (local × Friesian) cows with an average live weight of 404.9 ± 0.891 kg and milk yield (MY, averaged 16 ± 3.0 kg/h/d at the 7 wk) were used in a replicated 3 × 3 Latin square design with 28-d periods (including 8-d as an acclimatization phase). Cows were housed in individual tie stalls and body weight (BW) was recorded at the beginning of the study and then every week after morning lactating, to cover the amounts of concentrated feed prescribed. The three forms of ensiled RS (CGS, LSB, and HSB) were incorporated into three experimental diets, D1, D2, and D3, respectively. Between experimental cycles, all cows were fed a basal diet for 20 days to eliminate the influence of the previous treatment. Concentrate feed mixture (CFM) was provided at 2% of the body weight twice daily at a lactating time, while ensiled RS was provided *ad-libitum* after lactating, then removed two hours before milking for determining the amounts of consumed and refused of ensiled RS. The relative palatability ranking of experimental silage was determined using indexes calculated according to Obour and Oppong (2015) by dividing the daily consumption (weight) by the total weight of that silage offered and expressed as a percentage, where the potential maximum of 100 (highly palatable) and 0 (totally rejected), while the other classes stood, high (> 60%), medium (35–55%), and low palatability (< 25%). The milk yield (MY) of each cow was recorded twice at 6:00 a.m. and 05:00 p.m. weekly in each period, then composite morning and evening samples were stored at ^−^20 °C for chemical analysis. The percentage of 4% fat corrected milk (FCM) was calculated according to (Gaines 1928), and the yield of energy-corrected milk (ECM) was calculated by the formula of (Sjaunja et al. 1990) of each cow. During the last week of each period, a sample of diets and rectal feces was collected from each cow for 6 d twice daily after milking and kept refrigerated then composite (for each cow). Nutrient digestibility was estimated using the acid insoluble ash (AIA) technique, as described by Van Keulen and Young (1977; using 2 N HCl). Apparent digestibility was calculated from the concentrations of marker and nutrient in the feed and feces (% in dry matter) according to the following formula.

$$\begin{array}{l}{\rm{Apparent}}\,{\rm{Nutrient}}\,{\rm{Digestibility}}\,\left( {\rm{\% }} \right)\\{\rm{ = 100 \times (1 - }}\frac{{{\rm{marker}}\,{\rm{in}}\,{\rm{fee}}}}{{{\rm{marker}}\,{\rm{in}}\,{\rm{feces}}}}{\rm{ \times }}\frac{{{\rm{nutrient}}\,{\rm{in}}\,{\rm{feces}}}}{{{\rm{nutrient}}\,{\rm{in}}\,{\rm{feed/}}}}{\rm{)}}\end{array}$$

At the end of each experimental cycle, blood samples were withdrawn from the Jugular vein of each animal in the group at 8.00 am before feeding into heparinized vacutainers (Becton Dickinson, Rutherford, NY). Samples were centrifuged at 3000 rpm for 20 min to get supernatant then frozen at ^−^20 °C for the next analysis.

### Chemical analysis

#### Co-ensiling rice straw analysis procedures

The sensory analysis of ensiled RS (color, odor, texture, and moldiness) was inspected and subjectively judged by a panel involving five personnel exercised before commencing the actual evaluation, independently. Ensiled samples were extracted using a 50 g homogenized sample wet with 100 ml distilled water for 30 s at high speed in the blender (Mixed Countertop Blenders, Co., Fresh, Egypt), then filtered through double layers of cheesecloth. Silage pH was directly measured by using (HI-8424, HANNA Instruments, Woonsocket, RI, USA), then sampled for the microbial count. The remaining was processed to be stored at ^−^20 °C for analysis of fermentation products (NH_3_-N, lactic acid volatile fatty acids). The aerobic bacteria were counted on nutrient agar (Sangon Biotech Ltd., Shanghai, China), according to Zhang et al. (2016). As described by Ranjit and Kung (2000) the population of lactic acid bacteria was counted by plate count incubated at 37 °C for 48 h under anaerobic conditions, while the counting of yeasts and mold was done on a spread-plate of potato dextrose agar acidified with lactic acid (85%). The plates were incubated at 28 ºC, the counting of yeasts was done at 48 h, and the counting mold was done at 96 h. All the microbiological data were log-transformed. Filtrate ensiled RS samples (25 ml) were held to analyze lactic acid concentration by a method of James (1995), Ammonia-N (AOAC 2016), and total short-chain fatty acids (SCFA’s) according to Warner (1964). The molar proportions of VFA’s were determined by HPLC (column: Shodex RS Pak KC-811; Showa Denko K.K., Kawasaki, Japan; detector: DAD, 210 nm, SPD‐20 A; Shimadzu Co., Ltd, Kyoto, Japan; eluent: 3 mmol L^− 1^ HClO4, 1·0 mL min^− 1^; temperature: 50 °C). Dry matter recovery (DMR %) of tested ensiled RS was calculated using the equation of Dickerson et al. (1991).

$${\rm{DMR}}\,\left( {\rm{\% }} \right)\,{\rm{ = }}\,\left[ {\left( {{\rm{EMpo}}\,{\rm{x}}\,{\rm{DMpo}}} \right)\,{\rm{/}}\,\left( {{\rm{EMc}}\,{\rm{x}}\,{\rm{DMc}}} \right)} \right]\,{\rm{x}}\,{\rm{100,}}$$

where: DMR = dry matter recovery (%); EMpo = Ensiled mass at pile opening (kg); DMpo = dry matter at silo opening (%/100); EMc = Ensiled mass at closing (kg); and DMc = dry matter at closing (%/100).

#### Analyzing samples in-vivo study

Frozen samples of ensiled RS and feces were placed in aluminum trays and dried at 55 °C in an oven until constant weight and stored in poly-paper bags at 25 °C, in a dry and dark place, until chemical analysis. Subsequently, the materials of ensiled RS, feces, and CFM were grounded in a Wiley Mill (1-mm screen; Thomas Scientific, Philadelphia, PA, USA). the chemically analyzed EE, and ash as described by AOAC (2016), exceptionally, Kjeldahl N was analyzed in fresh samples and calculated as Kjeldahl *N* × 6.25. The NDF content was determined with a heat-resistant amylase according to Van Soest et al. (1991) and expressed on ash‐free basis (aNDFom), while the ADF content was expressed on ash‐free basis (ADFom). The total carbohydrates (TCH) were deduced with the following equation (100- (CP% + EE% + MM%)), and the non-fibre carbohydrates(NFC) from the difference between TCH and NDF (Sniffen et al. 1992). Milk samples were analyzed for content of fat, protein, lactose; solid not fat (SNF), and total solids (TS) by milk SCAN 133 BN Foss Electric, Denmark. Total protein (REF-30 115), Albumin, (REF-20 050), blood urea nitrogen (BUN, E-BC-K183-M), creatinine (REF-30 421), glucose, (REF-61 269), alanine transaminase (ALT, REF-22 226), and aspartate transaminase (AST, REF-22 226) were determined colorimetrically (Jenway 6300 Spectrophotometer U.K.) using the commercial kits (Bio Merieux 69,280 Marcy-1, Etoile, France®). Serum globulin was inferred by subtracting the albumin value from the total protein concentration.

### Statistical procedures

A simultaneous three Latin squares design was used for statistical analysis. Averages for each period and treatment combination were analysed to determine fixed effects of a square, period (P), diet (T), and their interaction (T×P), and random effects of cow within a square using mixed models procedures of SAS (version 9.1).

$${Y_{ijkl}} = {\bf{\mu }} + {{\bf{B}}_i} + {{\bf{P}}_j} + {{\bf{T}}_k}{\left( {\bf{B}} \right)_i} + {{\bf{D}}_l} + {{\bf{\varepsilon }}_{ijkl}}$$

where **Y**_ijkl_ is the dependent variable,μ is the mean of all observations, **B**_i_ is the fixed effect of block i, **P**_j_ is the fixed effect of period j, **T**_k_(B)_i_ is the random effect of diet k within block i, **D**_l_ is the fixed effect of diet l, and **ε**_ijkl_ is the normally distributed random residual error with an expected mean (0, σ^2^). The least-square means for all parameters were evaluated and differences were considered significant at *p* ≤ 0.05.

## Results

### Silage quality and chemical composition of diets

Sensory tests of ensiled RS are displayed in Table 1, where the highest quality with a yellow color and pleasant odor, the texture was loose and non-viscous. The results implied similarity among the different types of tested silage. The WSC content of ensiled RS was comparable for CGS, LSB, and HSB. Moreover, the results of fermentation characteristics showed that the values of pH, lactic acid (%DM), and NH_3_-N (%DM) were very close among the tested silage types. The total count of lactic acid bacteria, yeast, and aerobic bacteria is quite close and negligible. During the preservation period, no visible mold was observed in the experimental piles of CGS, LSB, and HSB.

**Table 1:** Ensiling quality parameters, and attributes of different types of tried silage

Items	Experimental Silage		
	CGS	LSB	HSB
Color	yellowish	yellowish	Light yellow
Odor	pleasant	pleasant	pleasant
Texture	Loose and non-viscous	Loose and non-viscous	Loose and non-viscous
**Fermentation characteristics**			
WSC, (%)	5.27	5.71	5.99
pH	3.87	3.94	3.98
Lactic acid, (%DM)	4.69	4.76	4.89
NH_3_-N/Total N	0.17	0.14	0.11
**Microbiology, (log**_**10**_ **cfu /g DM)**			
Lactic acid bacteria	7.81	7.86	7.93
Molds	ND	ND	ND
Yeast	2.25	2.11	2.04
Aerobic bacteria	5.34	5.21	5.03

CGS = ensiled rice straw including corn grains, LSB = ensiled rice straw including a low level of sugar beet, HSB = ensiled rice straw including a high level of sugar beet, and WSC = water-soluble carbohydrates, ND = not detected

Results of Table (2) show the chemical composition of different types of tested silage and experimental diets. Regarding tested silages, minor differences were established in the chemical analysis where aNDFom and ADFom slightly elevated with an increased incorporation level of SB in ensiled RS, while the contents of EE, CP, TCH, and NFC % decreased. The contents of OM, EE, TCH, and NFC in experimental diets that included either D2 or D3 decreased (*P* < 0.05) significantly, while aNDFom and ADFom proportions elevated (*P* < 0.05) significantly compared to a diet that includes CGS. The content of EE, NFC, or TCH was significantly (*P* < 0.05) decreased in a diet D3, or D1. DMR diminished slightly with the incorporated rise of SB to experimental silage.

**Table 2:** Chemical composition (% based on DM) of the tested silage, concentrate feed mixture, and experimental diets calculated according to lactating cows’ consumption

Items	Experimental Silage			CFM	Experimental Diets			SEM	*P*-value	
	CGS	LSB	HSB		D1	D2	D3		T	*P*
DM	40.70	39.28	38.37	91.42	58.63^a^	56.86^b^	55.79^c^	0.026	< 0.01	1.00
OM	85.97	85.27	84.92	92.87	89.77^a^	89.39^b^	89.20^c^	0.003	< 0.01	1.00
CP	7.52	7.48	7.45	15.89	12.04^b^	11.99^c^	12.27^b^	0.004	< 0.01	1.00
EE	1.44	1.33	1.21	3.05	2.32^a^	2.26^b^	2.20^c^	0.001	< 0.01	1.00
aNDFom	62.04	62.78	62.94	17.95	37.74^c^	38.48^b^	38.73^a^	0.023	< 0.01	1.00
ADFom	42.9	43.05	43.54	11.29	25.48^c^	25.84^b^	26.18^a^	0.017	< 0.01	1.00
TCH	77.01	76.49	76.43	73.93	75.31^a^	75.09^b^	75.01^c^	0.002	< 0.01	1.00
NFC	14.97	13.68	13.32	55.98	37.57^a^	36.61^b^	36.28^c^	0.021	< 0.01	1.00
DMR (%)	88.61	87.02	85.18	--	--	--	--	--	--	--

^a, b and c^ Within rows mean bearing different superscripts differ significantly at P ≤ 0.05

CGS = ensiled rice straw including corn grains, LSB = ensiled rice straw including a low level of sugar beet, HSB = ensiled rice straw including a high level of sugar beet, CFM = concentrate feed mixture consisted of (45% yellow corn, 6% soyameal, 25.5% wheat bran, 5% undecorticated cotton seed meal, 10% soybean meal, 5% molasses, 2% limestone, 1% salt, and 0.5% premix), D1 = control diet included CGS, D2 = diet included LSB, and D3 = diet included HSB

aNDFom = neutral detergent fiber, ADFom = acid detergent fiber, TCH = total carbohydrates(= 100-(CP + EE + Ash)), NFC = from the difference between TCH and NDF, DMR = dry matter recovery, T = treatments and P = period

### Feed intake, and animals performance

The data in Table 3 shows the average BW of the animals was similar among the different groups. The silage refused is represent 6.94, 3.44, and 1.94% of the feed offered while representing 7.46, 3.56, and 1.98% of feed consumed for CGS, LSB, and HSB, respectively. The amount of rejected silage was reflected in the silage consumed as shown in the results of Table 3, where the HSB and LSB consumed were significantly (*P* < 0.05) higher than the consumption of CGS. The amount of HSB consumed was high (*P* < 0.05) significant compared to consumed of the LSB. The palatability (%) of tested silage showed the same trend as silage consumption. The total feed intake of the experimental diets was significantly (*P* < 0.05) higher with fed diets that contained either HSB or LSB compared to CGS. At the same time, the total feed intake of the diet that contained HSB was significantly (*P* < 0.05) higher compared to a diet containing LSB.

**Table 3:** Bodyweight, feed intake, digestibility, and nutritive values of different experimental diets of lactating cows fed experimental silages

Items	Experimental Diets			SEM	*P*-value		
	D1	D2	D3		T	*P*	T*P
**Bodyweight**							
Live body weight, (kg /day)	404.71	404.90	405.22	0.891	0.92	0.08	0.86
**Feed intake**							
Silage offered, (kg /day)	6.48	6.48	6.48	---	---	---	---
Silage consumption, (kg /day)	6.03^c^	6.26^b^	6.36^a^	0.016	< 0.01	0.15	0.90
Relative palatability, (%)	93.07^c^	96.56^b^	98.06^a^	0.197	< 0.01	1.00	1.00
CFM, (kg /day)	7.41	7.40	7.41	0.016	0.92	0.08	0.87
Total feed intake, (kg /day)	13.43^c^	13.66^b^	13.77^a^	0.029	< 0.01	0.07	0.86
**Digestion coefficients (%)**							
DM	67.97^b^	68.61^ab^	69.26^a^	0.372	0.09	0.59	0.59
OM	70.69^c^	72.61^b^	73.19^a^	0.187	< 0.01	0.57	0.29
CP	64.34	64.52	64.69	0.216	0.54	0.53	0.55
EE	75.27	75.25	75.23	0.318	1.00	0.74	0.74
aNDFom	65.36^c^	67.93^b^	69.64^a^	0.250	< 0.01	0.06	0.09
ADFom	55.36^c^	57.18^b^	58.45^a^	0.299	< 0.01	0.84	0.84
TDN	65.65^b^	67.03^a^	67.35^a^	0.166	< 0.01	0.58	0.30
DCP	8.55^a^	8.28^b^	8.27^b^	0.028	< 0.01	0.50	0.53

^a, b and c^ Within rows mean bearing different superscripts differ significantly at *P* ≤ 0.05

CGS = ensiled rice straw including corn grains, LSB = ensiled rice straw including a low level of sugar beet, HSB = ensiled rice straw including a high level of sugar beet, CFM = Concentrate feed mixture, D1 = control diet included CGS, D2 = diet included LSB, and D3 = diet included HSB. aNDFom = neutral detergent fiber, ADFom = acid detergent fiber

TDN (%) = DOM%+1.25 × (DEE%), DCP = digestibility coefficient x CP, T = treatments and P = period

The digestion coefficient of DM and OM was significantly (*P* < 0.05) higher with fed a D3 compared to D1. There was no significant (*P* > 0.05) difference among groups in CP and EE digestibility. The digestibility coefficients of aNDFom and ADFom were significantly (*P* < 0.05) increased with fed diets D2 and D3 compared to that D1, nevertheless, the digestibility was increased (*P* < 0.05) with fed diets containing HSB than that containing LSB. Feeding a diet containing LSB or HSB led to a significant (*P* < 0.05) increase in TDN value, compared to those containing CGS, while no significant (*P* < 0.05) differences were found in TDN values between the diets containing LSB or HSB. The DCP values of a diet containing LSB or HSB significantly (*P* < 0.05) decreased compared to that containing CGS, and the values in the diet containing HSB were significantly (*P* < 0.05) lower than those that contained LSB.

### Animal performance and blood constituents

Data in Table 4 presented MY and composition, and FCR of different tested diets. The values of MY, FCM, and ECM for fed cows a D2 did not differ compared to those fed on D1, except that the ECM values were significantly (*P* < 0.05) lower for feeding D1. Milk fat was significantly (*P* < 0.05) higher with fed cows D3 compared to those fed D1, while no significant difference was found when fed a diet containing D2 compared to a diet containing D3. Feeding cows on a diet containing LSB or HSB led to an increase in the milk content of protein, lactose, total solids, and SNF, except for lactose content with the control diet, and there was an interaction effect for milk fat and total solids. The same contents were (*P* < 0.05) increased when fed cows a diet containing HSB compared to those fed on LSB. The values of FCR which are expressed as kg DM/kg FCM and kg TDN/kg FCM reduced (*P* < 0.05) significantly when fed cows on D3 compared to when fed D1 or D2, respectively.

**Table 4:** Milk yield, its composition, and feed conversion ratio of different experimental diets of lactating cows fed experimental silages

Items	Experimental Diets			SEM	*P*-value		
	D1	D2	D3		T	*P*	T*P
**Milk production (kg /h /d)**							
Milk yield, (MY)	16.66^b^	17.05^b^	18.00^a^	0.305	0.02	0.13	0.71
Fat corrected milk, (FCM)	14.43^b^	15.21^b^	16.46^a^	0.308	< 0.01	0.75	0.49
Energy corrected milk, (ECM)	13.78^c^	14.73^b^	16.34^a^	0.287	< 0.01	0.58	0.63
**Milk composition (%)**							
Fat	3.12^b^	3.27^ab^	3.43^a^	0.053	< 0.01	0.09	0.016
Protein	2.56^c^	2.78^b^	3.12^a^	0.056	< 0.01	0.65	0.88
Lactose	4.34^b^	4.45^b^	5.13^a^	0.051	< 0.01	0.11	0.85
Total solid	10.79^c^	11.20^b^	12.38^a^	0.056	< 0.01	< 0.01	< 0.01
Solid not fat	7.68^c^	7.94^b^	8.95^a^	0.083	< 0.01	0.47	0.94
**Feed conversion ratio**							
kg DM intake/kg FCM	0.933^a^	0.904^a^	0.840^b^	0.019	< 0.01	0.82	0.50
kg TDN intake /kg FCM	0.614^a^	0.608^a^	0.566^b^	0.013	0.04	0.78	0.41

^a, b and c^ Within rows mean bearing different superscripts differ significantly at *P* ≤ 0.05

CGS = ensiled rice straw including corn grains, LSB = ensiled rice straw including a low level of sugar beet, HSB = ensiled rice straw including a high level of sugar beet, D1 = control diet included CGS, D2 = diet included LSB, and D3 = diet included HSB., and TDN = total digestible nutrients, T = treatments and P = period

As shown in Table 5, blood constituents of TP, albumin, globulin, AST, ALT, and urea nitrogen were not different (*P* < 0.05) significantly among cows groups that were fed diets containing different forms of ensiled RS. The level of glucose in the cow’s blood increased (*P* < 0.05) significantly with the diets containing LSB and HSB compared to the control diet containing CGS. In addition, the level of glucose in the blood increased (*P* < 0.05) significantly when feeding cows on diets that contained HSB compared to those that contained LSB.

**Table 5:** Biochemical blood parameters of lactating cows fed different experimental diets

Items	Experimental Diets			SEM	*P*-value		
	D1	D2	D3		T	*P*	T*P
Total protein, (g /dL)	6.19	6.18	6.19	0.017	0.80	0.66	0.92
Albumin, (g /dL)	3.25	3.25	3.24	0.084	1.00	0.92	0.61
Globulin, (g /dL)	2.94	2.93	2.95	0.070	0.98	0.93	0.54
AST, (IU/ L)	86.92	86.84	86.93	0.598	0.99	1.00	1.00
ALT, (IU /L)	19.22	19.17	19.22	0.162	0.97	0.40	0.30
BUN, (mg /dL)	16.73	16.54	16.51	0.158	0.59	0.75	0.73
Glucose, (mg /dL)	65.40^c^	67.41^b^	71.13^a^	0.194	< 0.01	1.00	1.00

^a, b and c^ Within rows mean bearing different superscripts differ significantly at *P* ≤ 0.05

CGS = ensiled rice straw including corn grains, LSB = ensiled rice straw including a low level of sugar beet, HSB = ensiled rice straw including a high level of sugar beet, D1 = control diet included CGS, D2 = diet included LSB, and D3 = diet included HSB. AST = Aspartate aminotransferase, ALT = Alanine aminotransferase, BUN = blood urea nitrogen, T = treatments and P = period

## Discussion

Sensory evaluations such as color, odor, and texture of tested silage were complete similarities among the different types of ensiled RS and consistent with well-fermented silage characteristics as recommended by Oladosu et al. (2016). The three silages tested are high-quality, the content of WSC in the ensiled RS confirms fermentation quality. Ni et al. (2018) instructed that the optimal concentration of WSC should be 50 g/kg DM content. All experimental silages had similar pH values (ranged 3.87 to 3.98) and thus classified as very good silage (Oladosu et al. 2016). The final pH of silage is affected by many factors but is most related to the concentration of lactic acid and buffering capacity of the crop (Ni et al. 2018). These results were consistent with Jahanzad et al. (2014) who concluded that the pH value in silage was conjugated with the content of WSC and N levels. Previous studies by Beauchemin (2006) noted that the ammonia-N level decrease with an increased level of SB in silage, these results are compatible with the results of the present study. Oladosu et al. (2016) attributed that an ammonia-N level in silage might be influenced by the activity of polyphenol oxidase. The silage of HSB contained a higher population of LAB than other silages. Concomitantly with a slight increase in LAB count, a little decrease in the count of yeast and aerobic bacteria was shown particularly with increased incorporation of SB, which is consistent with the fact that lactic acid bacteria can inhibit other microorganisms (Abedo 2006).

The slight differences in some nutrients among experimental silage mixtures may be attributed to the change in the chemical composition of the ingredients and additives. The results of the present study were coherent with typical values reported by Gurbuz and Kaplan (2008); Beauchemin (2006) concerning the values of DM, OM, CP, EE, NFC, and TCH contents of sugar beet silage. The slight content of aNDFom and ADFom for ensiled RS included SB compared with the CG is highly consistent with a previous statement by Gurbuz and Kaplan (2008) when incorporating SB with stalks of a corn hybrid. Further, the higher content of ash in HSB and LSB compared with CGS is presumably due to the high ash content of the SB plants and/ or to some soil contamination during harvesting (Evans and Messerschmidt 2017). Differences in DM intake due to the palatability and nutritional value of the experimental diets could be reflected in increased content of DM, OM, EE, NFC, and TCH with D1 compared to D2 and D3, as well as increased contents of aNDFom, ADFom, and ash in D3 compared with D1.

The highest values of silage consumption and lowest values of silage refused may attribute to higher palatability as shown in Table 3. A similar effect is communicated by Evans and Messerschmidt (2017) who suggest that DMI for lactating cows increased with feeding SB. A higher DMI of the D3 than that of D2 might show that high-level incorporation of SB in ensiled RS was preferable to low-level because ruminants generally prefer sweet-flavored forages (Gurbuz and Kaplan 2008). The improvement in DMI for the D2 and D3 diets containing silage incorporated with SB, compared to D1 may be due to the improvement in the digestion parameters of these tested diets, as indicated by the results of Table 3, whose indices agree with the results of Beauchemin (2006).

The higher digestibility coefficient of aNDFom and ADFom with diet D3 which included HSB may be due to higher of ruminal degradability, also results shown in Table 1 indicate that non-structural carbohydrates in the diet did not reduce rumen pH and this is consistent with the results of Clark and Armentano (1997). Moreover, Abedo (2006) stated that the higher aNDFom and ADFom digestibility may be due to that pectin fermentation results in less lactate and a higher acetate-to-propionate ratio without affecting the digestibility of cellulose and hemicellulose. In addition, the high digestibility coefficient of some nutrients in D3 compared to D1 could be interpreted by three explanations: (1) the decrease in the outflow rate of feed in the rumen because of their higher structural carbohydrates contents, especially pectin substances (Poorkasegaran and Yansari 2014). (2) the content of NDF in sugar beet is considered unique because it has been shown to feature a very high cation exchange capacity (Evans and Messerschmidt 2017), which tends to promote pH stewardship and a more stable rumen environment. (3) by the SB’s ability to increase the efficiency of utilization of various components of diet and microbial N synthesis (Oladosu et al. 2016) due to their suitable protein and energy contents which made it completely available in the gastrointestinal tract (Clark and Armentano 1997).

Similarly, Sorathiya et al. (2015) concluded that the aNDFom in SB has a higher digestibility rate as a partial replacer to green fodder. Morover, Abedo (2006) showed that DM, OM, CP, and CF digestibility and nutritive value as TDN increased with using dried SB pulp in animal diet. Furthermore, Suliman et al. (2013) showed the best TDN or DCP values achieved for a diet containing concentrate feed mixture plus SB tops silage compared with the control diet which contains CFM plus berseem hay.

Increased milk production, fat-corrected milk, and energy-corrected milk for cows fed D3 compared with fed on either D2 or D1 may be attributed to higher DMI and this is consistent with the results of (Clark and Armentano 1997). In a study, Sorathiya et al. (2015) confirmed an increase in daily MY with no significant difference between cows fed SB or when partially replaced with green fodder. The high values of milk fat and other milk parameters with a diet containing ensiled RS included SB could be due to the relative enough protein input and the high content of neutral detergent soluble fibre, chiefly pectin. Abedo (2006) revealed that fermentation of pectin produces less lactate and more acetate to propionate ratio in the rumen.

The values of FCR as kg DM/kg FCM and kg TDN/kg FCM in cows fed diet D3 were 9.99 and 7.94% respectively better, with significantly different (*P* < 0.05) than cows fed the D1 diet. This improvement in FCR with HSB is related to the improvement of MY production. These results agree with Abedo (2006) that FCR was significantly higher for lambs fed a diet that contained dry SB pulp compared with the other diets.

The values of total protein, albumin, globulin, and urea remained unchanged and were within the normal ranges reported in the reference values by Radostitis et al. (2000). Moreover, The means values of AST and ALT obtained were within the normal activity range recorded in the blood (78–132 U/L and 11–40 U/L., respectively) for healthy cattle by Ingvartsen (2006); Silanikove and Tiomkin (2010). Per contra, the mean values of glucose in group D3 and D2 of cows were much higher and statistically significant compared with the control group D1, this might attribute to SB plants, which have more readily hydrolysable sugar content than CG (Oladosu, et al. 2016). The plasma glucose values found are contrary to the previously obtained values in lactating dairy cows by Gurbuz and Coskun (2011) which decreased with SB content. The absence of significant differences in the blood metabolites of the experimental animals indicates a similarity in terms of the quality and quantity of diet (Ndlovu et al. 2009), thus all animals had the same good health and nutritional status.

## Conclusion

In conclusion, co-ensiling rice straw with the whole plant of sugar beet leads to induce a good fermentation that causes acceptable to animals on it, consequently improving consumption. Sugar beet addition contributes to overcoming the lack of soluble carbohydrates in rice straw, which affects ensiling fermentation. Co-ensiling whole sugar beet and rice straw as an agricultural residue is a good approach to improve the digestive processes and thus increase the nutritional value of rice straw without negative effects on animal performance.

## Data Availability

The data that support the findings of this study are available from the corresponding author upon reasonable request.
